# Countdown to 2015 country case studies: what have we learned about processes and progress towards MDGs 4 and 5?

**DOI:** 10.1186/s12889-016-3401-6

**Published:** 2016-09-12

**Authors:** Corrina Moucheraud, Helen Owen, Neha S. Singh, Courtney Kuonin Ng, Jennifer Requejo, Joy E. Lawn, Peter Berman, the Countdown Case Study Collaboration Group, Ahmed Salehi, Zhou Hong, Carine Ronsmans, Gao Yanqiu, Hillena Kebede, Carlyn Mann, Jenny Ruducha, Mekonnen Tadesse, Anthony Ngugi, Emily Keats, William Macharia, Nirmala Ravishankar, Neha Singh, John Tole, Jennifer Bryce, Tim Colbourn, Bernadette Daelmans, Mercy Kanyuka, Humphreys Nsona, Nadia Askeer, Zulfiqar Bhutta, Zaid Bhatti, Arjumand Rizvi, Jessica Niño de Guzman, Luis Huicho, Cesar Victora, Hoviyeh Afnan-Holmes, Theopista John, Joy Lawn, Moke Magoma, Georgina Msemo

**Affiliations:** 1 University of California Fielding School of Public Health, Los Angeles, CA 90095 USA; 2 Centre for Maternal, Adolescent, Reproductive & Child Health, London School of Hygiene & Tropical Medicine, London, WC1E 7HT UK; 3 Harvard T.H. Chan School of Public Health, Boston, MA 02115 USA; 4 Institute for International Programs, Johns Hopkins Bloomberg School of Public Health, Baltimore, MD USA

**Keywords:** Millennium Development Goals, Maternal health, Neonatal health, Child health, Reproductive health, Coverage, Equity, Health systems, Health finance, Accountability

## Abstract

**Background:**

Countdown to 2015 was a multi-institution consortium tracking progress towards Millennium Development Goals (MDGs) 4 and 5. Case studies to explore factors contributing to progress (or lack of progress) in reproductive, maternal, newborn and child health (RMNCH) were undertaken in: Afghanistan, Bangladesh, China, Ethiopia, Kenya, Malawi, Niger, Pakistan, Peru, and Tanzania. This paper aims to identify cross-cutting themes on how and why these countries achieved or did not achieve MDG progress.

**Methods:**

Applying a standard evaluation framework, analyses of impact, coverage and equity were undertaken, including a mixed methods analysis of how these were influenced by national context and coverage determinants (including health systems, policies and financing).

**Results:**

The majority (7/10) of case study countries met MDG-4 with over two-thirds reduction in child mortality, but none met MDG-5a for 75 % reduction in maternal mortality, although six countries achieved >75 % of this target. None achieved MDG-5b regarding reproductive health. Rates of reduction in neonatal mortality were half or less that for post-neonatal child mortality. Coverage increased most for interventions administered at lower levels of the health system (e.g., immunisation, insecticide treated nets), and these experienced substantial political and financial support. These interventions were associated with ~30–40 % of child lives saved in 2012 compared to 2000, in Ethiopia, Malawi, Peru and Tanzania. Intrapartum care for mothers and newborns -- which require higher-level health workers, more infrastructure, and increased community engagement -- showed variable increases in coverage, and persistent equity gaps. Countries have explored different approaches to address these problems, including shifting interventions to the community setting and tasks to lower-level health workers.

**Conclusions:**

These Countdown case studies underline the importance of consistent national investment and global attention for achieving improvements in RMNCH. Interventions with major global investments achieved higher levels of coverage, reduced equity gaps and improvements in associated health outcomes. Given many competing priorities for the Sustainable Development Goals era, it is essential to maintain attention to the unfinished RMNCH agenda, particularly health systems improvements for maternal and neonatal outcomes where progress has been slower, and to invest in data collection for monitoring progress and for rigorous analyses of how progress is achieved in different contexts.

**Electronic supplementary material:**

The online version of this article (doi:10.1186/s12889-016-3401-6) contains supplementary material, which is available to authorized users.

## Background

*The Millennium Developm*ent *Goals* (MDGs) period concluded in 2015, and a plethora of reports were released to assess progress made. MDGs 4 and 5 were at the heart of the health-related MDGs. MDG 4 called for a reduction of childhood (under age 5) mortality by two-thirds, and MDG 5 focused on the improvement of maternal health through a reduction of maternal mortality by three-quarters and a later addition of MDG-5b regarding universal access to reproductive health [1]. Although maternal and child mortality have been reduced by almost 50 % since the 1990s [2], progress is varied across and within countries, and some aspects – such as newborn survival and reproductive health – received less attention until recently and have seen slower progress [3]. In addition to varied progress between different outcomes, there are major differences in progress between countries, even neighbouring countries and understanding these differences is key to informing future progress.

Countdown to 2015 (Countdown) was established in 2005 as a multi-disciplinary, multi-institutional collaboration to track progress towards MDGs 4 and 5 in the 75 countries where more than 95 % of all maternal, newborn and child deaths occur. Countdown uses country-specific data to stimulate and support country progress, to promote accountability of governments and development partners, to identify knowledge gaps, and to propose new actions to reduce newborn and child mortality and improve maternal health [1].

**Fig. 1:**
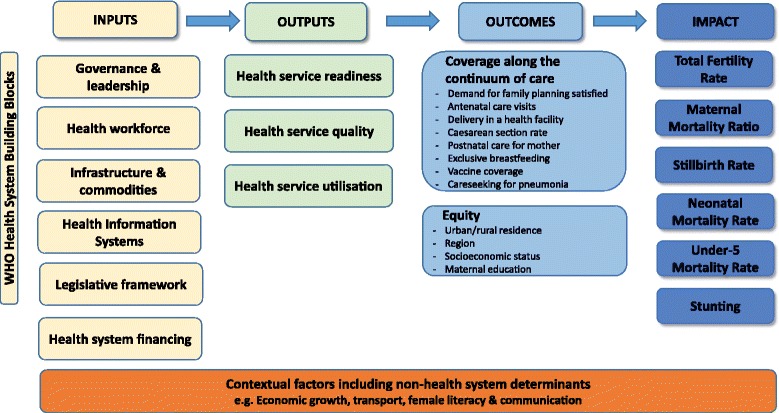
Evaluation framework for Countdown to 2015 country case studies. Source: Afnan-Holmes et al. [11]

To complement its global monitoring effort, Countdown undertook in-depth country case studies to improve understanding of the causes and processes that underpinned or detracted from achievement of MDGs 4 and 5. A secondary aim of the case studies was to strengthen country-level capacity to conduct research, and to monitor progress in reproductive, maternal, newborn and child health (RMNCH) within countries. Countdown country case studies were led by national investigators with support from the global Countdown team and from Countdown’s four technical working groups: coverage, equity, health systems and policies, and financing. This work drew upon Countdown’s approach of linking changes in health outcomes to changes in intervention coverage and key coverage determinants, such as equity, policies and systems, and financing. The standard Countdown evaluation framework is displayed in Fig. 1 (supplementary information on the evaluation framework and analyses is available in Additional file 1).

**Fig. 2:**
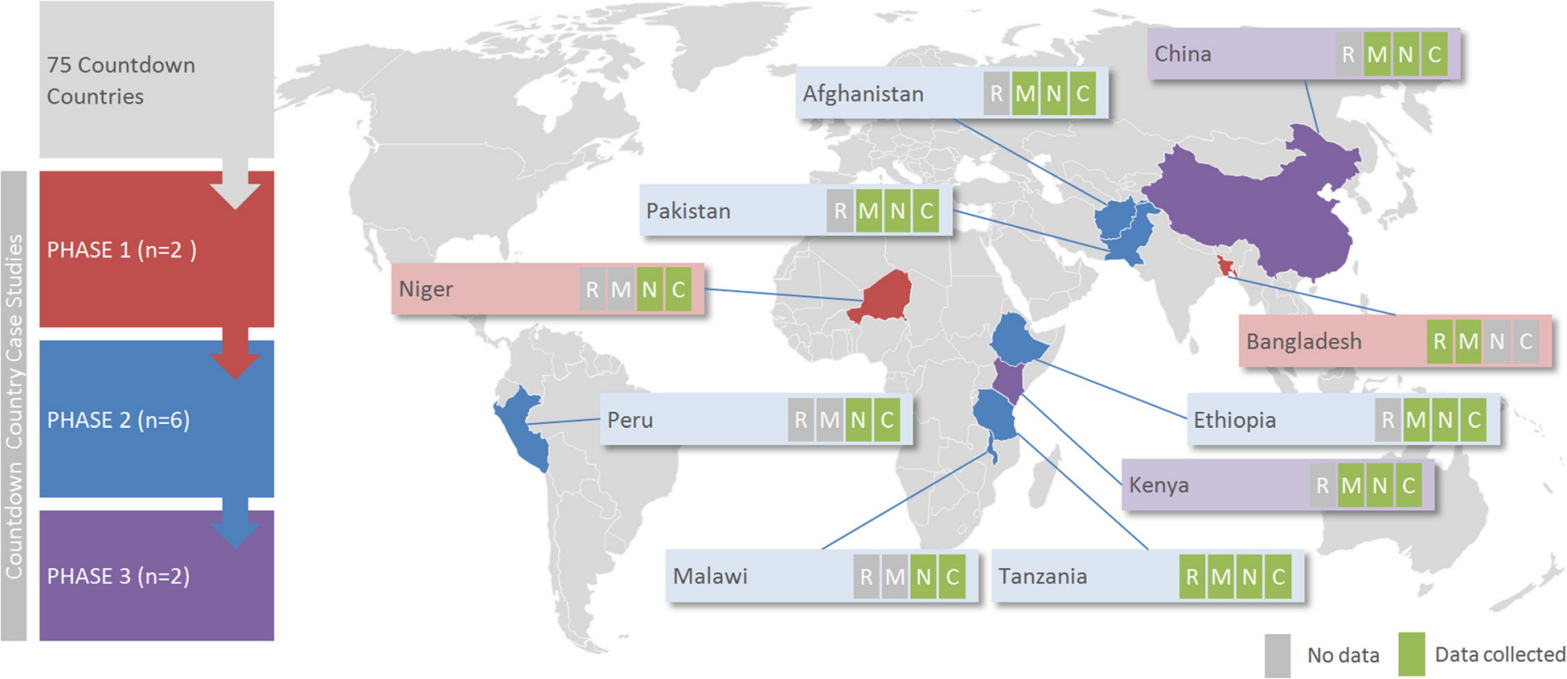
Overview of the case study country selection, geography and focus along the continuum of care accounting to R (reproductive), M (maternal), N (newborn) and C (child) health

The first set of case studies (phase 1), carried out in Niger and Bangladesh, were published in The Lancet in 2012 and 2014 respectively [4, 5] and contributed to the development of a standardised analysis approach that has been applied in subsequent case studies. A second phase of case studies was undertaken in Afghanistan [6], Ethiopia [7], Malawi [8], Pakistan [9], Peru [10], and Tanzania [11]. China and Kenya (phase 3) were added later (Fig. 2) (further details on the case studies are provided in the Additional file 1).

The objectives of this paper are to:

1. Compare quantitative data to evaluate MDG 4 and 5 progress, and changes in coverage, equity and national context, in the case study countries (depending on data availability per indicator): Afghanistan, Bangladesh, China, Ethiopia, Kenya, Malawi, Niger, Pakistan, Peru, and Tanzania.
2. Use content analysis methods to explore factors that may have enabled or hindered progress towards achieving MDGs 4 and 5 across the six countries with publicly available case study results at the time of publication: Afghanistan, Ethiopia, Malawi, Pakistan, Peru, and Tanzania.

## Methods

For this cross-cutting analysis, all case study materials – including reports, manuscripts, papers and presentations from each team and from three capacity building workshops (details on these workshops are available at (http://www.countdown2015mnch.org) [12] – were reviewed by study authors to identify factors leading to and detracting from progress on MDGs 4 and 5. We consulted with experts from each of the Countdown technical working groups as well as the case study teams to validate our findings. More details on the methodologies are presented below, and in the Additional file 1. Figure 2 presents an overview of the case study countries, including their geography and case study’s focus across the RMNCH continuum. Each country case study should be referred to for full detail about its findings and implications.

### Sample selection

The first two case study countries (Bangladesh and Niger) were selected based on data availability and existing strong partnerships between Countdown members and in-country research institutions. In response to substantial interest from other countries for similar analyses, Countdown pursued a portfolio of additional case studies. Nine of the 75 Countdown countries (selected based on data availability and non-duplication with other in-depth analyses) were asked to submit proposals; six country teams were ultimately selected in February 2013 to write full case studies (“phase 2”). Early in 2014, an additional nine countries submitted proposals, from which two additional case study teams were selected. (Further details on this process are available in Additional file 1: Figure B.1-2.)

### Objective 1: Compare quantitative data to evaluate MDG 4 and 5 progress, and changes in coverage, equity and national context

#### Analysis overview and objectives

Quantitative data on the Countdown case study countries were analysed across the evaluation framework (Fig. 1).^1^ The analysis aimed to assess the countries’ progress toward MDGs 4 and 5 by systematically evaluating trends since 1990 in impact indicators, coverage of key indicators across the RMNCH continuum of care (CoC), and changes in political, economic and social factors. Additionally, this analysis compared case study country results on the contribution of health intervention coverage to childhood mortality change since the year 2000. Each analysis included those case study countries with available data; Additional file 1: Table B.2-1 displays the representation of countries within the quantitative results presented in this paper.

#### Methodology

This cross-cutting analysis examined impacts, intervention coverage and equity, the role of intervention coverage change on mortality declines, and social and economic indicators. Data sources and methods are described in more detail in Additional file 1 section B.2. Data on impact indicators were obtained from the most recently published United Nations estimates at the time of this analysis [13–17]. Information on coverage and equity was obtained for select indicators recommended by the United Nations Commission on Information and Accountability (CoIA) for Women’s and Children’s Health from the 2015 Countdown report and database [18]. Changes per year for impact and coverage indicators were calculated using the standard formula for annual average rates of change. The Lives Saved Tool (LiST) was used to estimate how changes in the coverage of key interventions may be associated with mortality change at the national level; results from the countries’ own LiST analyses [7, 8, 10, 11, 19] are reported here. More detail on the LiST methodology overall can be found in the literature [20]. Data for the social and economic indicators investigated here are those utilised by the Maternal and Child Epidemiology Estimation group (omitting those that overlap with coverage, outcome or impact indicators otherwise investigated by the case study teams) [21].

### Objective 2: Undertake content analysis research to explore factors that may have enabled or hindered progress towards achieving MDGs 4 and 5

#### Analysis overview and objectives

A content analysis was undertaken of five of the “phase 2” case studies,^2^ to systematically identify the core themes emerging from the Countdown country case studies, based on the evaluation framework (Fig. 1) and the World Health Organisation (WHO) health systems building block model [22]: to explore how progress towards MDGs 4 and 5 was achieved (or not), by examining patterns in and relationships between coverage level and trends and key health systems and contextual factors.

#### Methodology

Two authors (HO, CN) independently reviewed all final case study manuscripts and reports and identified factors that hindered or enabled progress across the content areas in the evaluation framework (see Additional file 1) by the categories of reproductive health, maternal health, child health, and newborn health. All relevant information was manually extracted from the manuscripts, and organised by country into an Excel spreadsheet (Additional file 1 section B.3).

The collated information was then synthesised using the WHO health systems building block framework to identify similarities and differences across countries. The case studies only included comparable and pertinent information on five of the six input variables included in the WHO health system building blocks [22]: governance and leadership; health systems financing; health workforce; service delivery; and infrastructure and commodities (i.e., information systems was not included). Non-health sector factors posited by the teams as influencing health system functionality and health outcomes in their respective countries were also examined.

Results were then verified through consultation with the country teams. The principal investigators from each of the country teams were asked via email and a webinar to review the initial content analysis results and to confirm the validity (consistent with their understanding of their country’s experience) and comprehensiveness of the findings. Based on these consultations, the results were revised as relevant and finalised.

## Results

### Objective 1: Compare quantitative data to evaluate MDG 4 and 5 progress, and changes in coverage, equity and national context

#### Impact

**Fig. 3:**
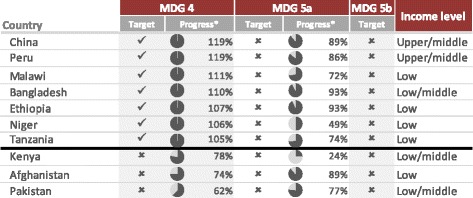
Countdown to 2015 country case study progress to achieving MDGs 4 and 5 by income level. Data sources: MDG reports 2015, income level from the World Bank 2015. *i.e., % achievement of 66 % reduction for MDG 4 and 75 % reduction for MDG 5a

All Countdown case study countries achieved reductions in fertility and all mortality indicators (neonatal mortality rate [NMR], under-5 mortality rate [U5MR], maternal mortality ratio [MMR]) over the full MDG period – although to varying degrees and with mixed progress on achieving the MDGs, as shown in Fig. 3. (Data are presented in Additional file 1: Table B.2-2.) The prevalence of stunting among children under age 5 also declined (in case study countries with available data, see Additional file 1: Table B.2-2), with average annual rates of reduction of 4.3 % in Peru, between 1.7 and 2.5 % in Bangladesh, Ethiopia, Malawi and Tanzania, and 0.6 % in Niger.

**Fig. 4:**
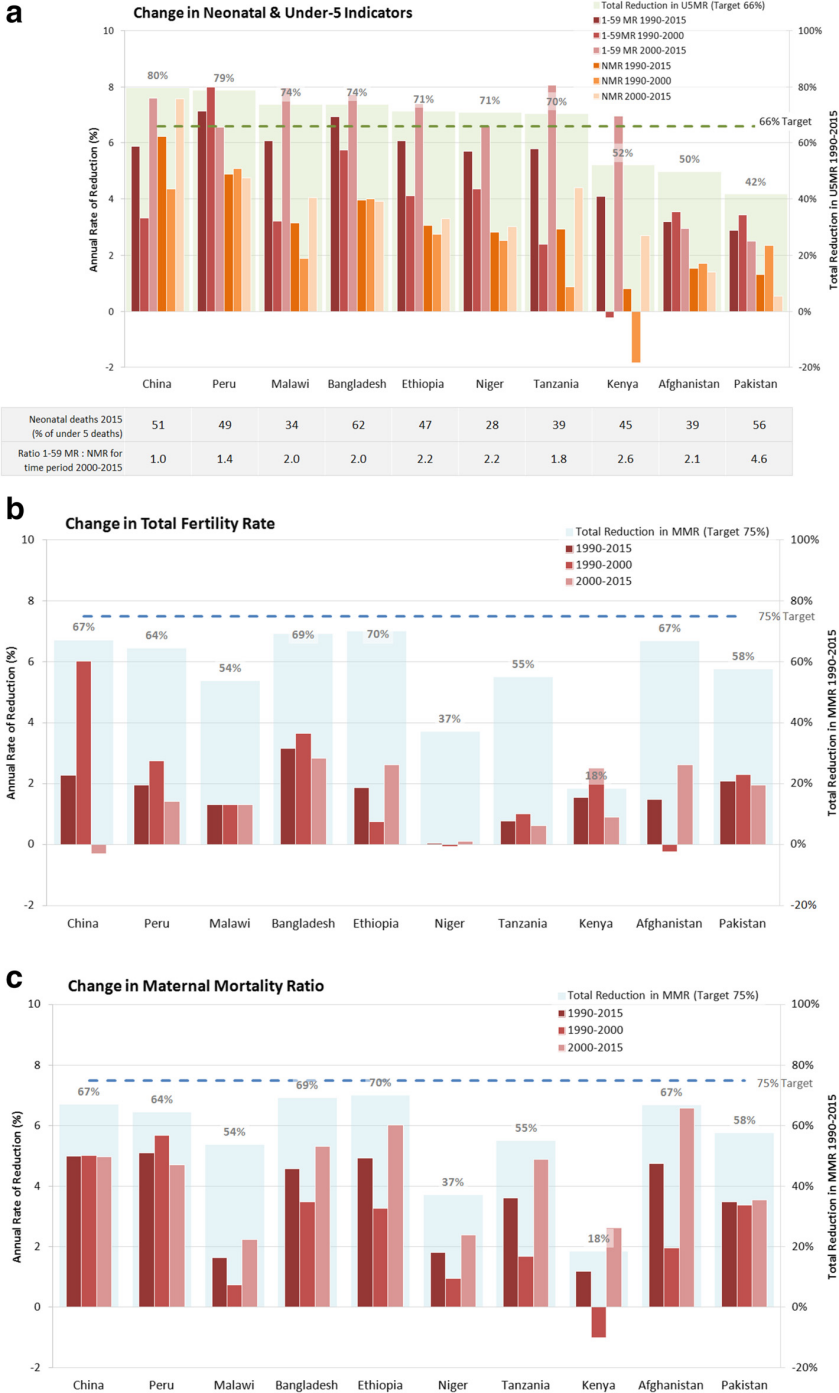
Annual rate of reduction in impact indicators, in each Countdown to 2015 case study country, for the full MDG period (1990–2015), as well as for each decade (1990–2000 and 2000–2015). **a** Change in Neonatal & Under-5 Indicators. **b** Change in Total Fertility Rate. **c** Change in Maternal Mortality Ratio. Data sources: Analysis from UN Interagency Group for Child Mortality Estimation (IGME) in 2015; United Nations Population Division. World Population Prospects (WPP): The 2015 Revision. Total Fertility (TFR); WHO. 2015. Levels and Trends for Maternal Mortality: 1990 to 2015. Geneva: World Health Organization

Figure 4 presents annual rates of change in the ten case study countries for neonatal, maternal, and childhood mortality, as well as total fertility rate, over the entire MDG period (1990–2015) and for each decade (1990–2000 and 2000–2015). The countries are presented – here and throughout – in descending order of U5MR reduction (1990–2015). The case study findings parallel those found across the 75 Countdown countries, where the largest reduction was observed in childhood mortality, and there were accelerated improvements post-2000 for many impact indicators. More details on the trends and findings for all of Countdown are available in the 2015 Countdown report [1].

In general, among the indicators studied, the Countdown case study countries achieved the most progress in reducing mortality among children aged 1–59 months: a 5.4 % average annual reduction since 1990, compared to 3.6 % for MMR and 3.1 % for NMR. Seven of the case study countries met, and even exceeded, MDG 4 to reduce their U5MR by two-thirds between 1990 and 2015: Bangladesh, China, Ethiopia, Malawi, Niger, Peru and Tanzania (Fig. 4a). These countries also reduced their NMR at approximately 3 % average annual reductions over this period which is more than their neighbours, but still half the rate of progress they made for child deaths after the neonatal period. In all countries the annual rate of reduction for NMR after the year 2000 was less than that for 1–59 month olds. In Pakistan neonatal deaths accounted for 56 % of under-5 deaths in 2015 and yet the annual rate of reduction for 1–59 month olds after the year 2000 is still 4.6 times higher than that for neonates. Progress in reducing mortality among neonates and children aged 1–59 months accelerated after the year 2000 in all case study countries except Afghanistan, Pakistan and Peru.

Fertility decline was slower post-2000 in many case study countries (Peru, Bangladesh, Tanzania, Kenya, and Pakistan) compared with before, and fertility increased in China after the year 2000 (Fig. 4b).

Although none of the case study countries met MDG 5, all reduced their MMR with six countries achieving >75 % progress toward the goal of 75 % reduction in MMR (with Bangladesh and Ethiopia achieving over 90 % progress) (Fig. 3). The most substantial annual reductions were seen in China, Ethiopia and Peru (approximately a 5.0 % annual rate of reduction), Afghanistan (4.8 %) and Bangladesh (4.6 %). Apart from Peru and China, all countries showed greater annual rates of reduction after the year 2000 (Fig. 4c).

#### Outcome - coverage

**Fig. 5:**
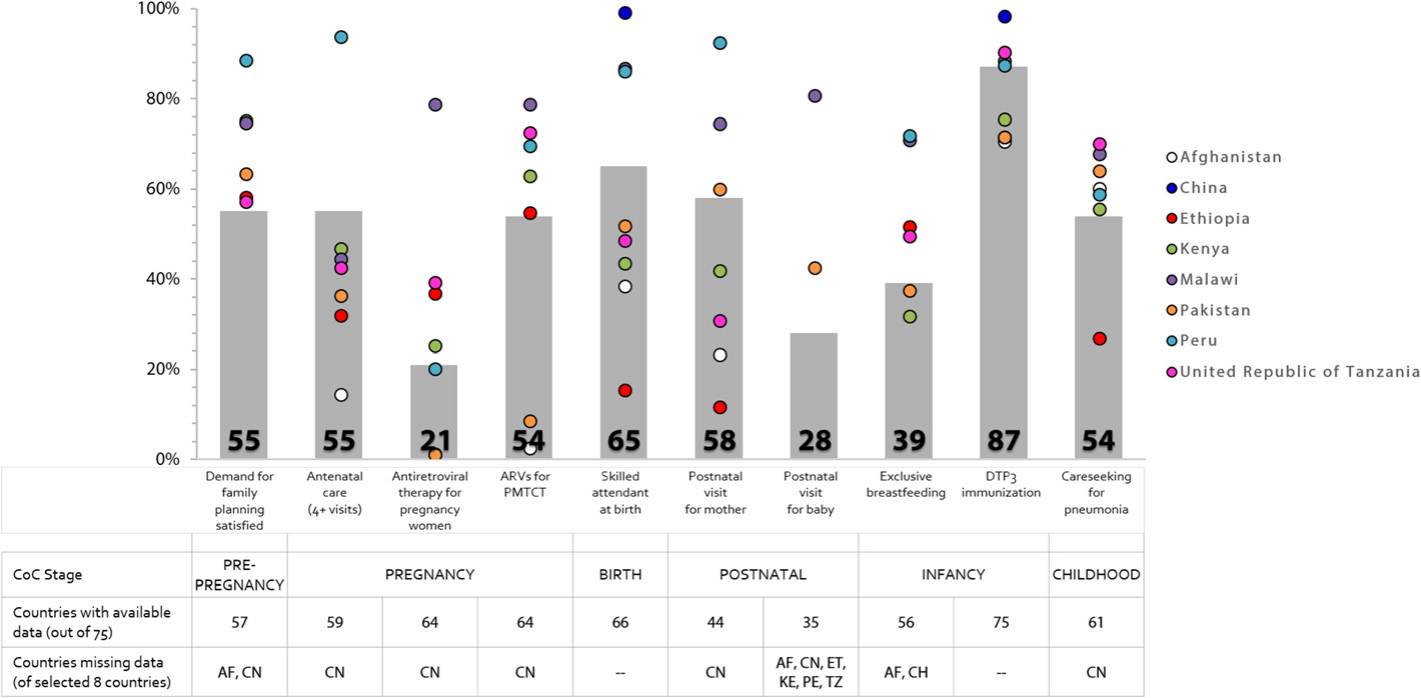
Most recent median national coverage (%) of selected Commission on Information and Accountability (CoIA) indicators in 75 Countdown to 2015 countries, with national coverage for case study countries. Grey bars indicate the median level of coverage per CoIA indicator across all 75 Countdown countries; dots represent the national level of coverage for each CoIA indicator per case study country

**Fig. 6:**
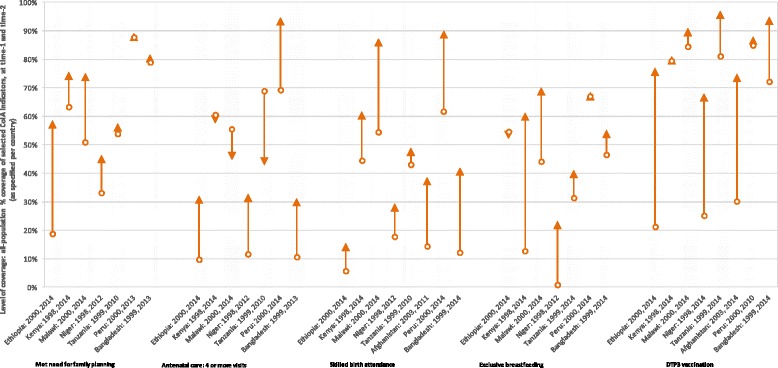
Change in coverage of select Commission on Information and Accountability (CoIA) indicators in Countdown to 2015 case study countries, over time. This figure includes only those case study countries with available data. Antenatal care and skilled birth attendance are reported among births during the 3 years preceding the survey

Figure 5 displays the most recent level of coverage for CoIA indicators at the time of publication, as a median value among all 75 Countdown countries and the national coverage for each case study country, and Fig. 6 displays change in these indicators since 1990 (for countries with available data). Countdown countries have attained rates of DTP3 (Diphtheria-tetanus-pertussis) immunisation that meet or exceed 70 % coverage, but this is the only indicator with such universally high coverage. Interventions during and after birth (e.g., skilled birth attendance [SBA] and postnatal care) have the largest ranges of coverage across the case study countries of 84 and 81 percentage points, respectively, followed by antenatal interventions (e.g., attendance at four or more antenatal visits has a range of 80 percentage points, and antiretrovirals during pregnancy and prevention of mother-to-child transmission of HIV have a range of 79 percentage points).

As shown in Fig. 6, all interventions saw increased coverage in the case study countries over this period – except attendance at four or more antenatal visits, which decreased in Kenya, Malawi and Tanzania (but increased in Bangladesh, Ethiopia, Niger and Peru); and exclusive breastfeeding in Ethiopia which declined over the period. Skilled birth attendance coverage more than tripled in Afghanistan, Bangladesh and Ethiopia; DTP3 vaccination increased by a similar degree in Afghanistan, Ethiopia, and Niger. Ethiopia also saw a large increase in demand satisfied for family planning (from 19 to 59 %), and Niger experienced a very large increase in the prevalence of exclusive breastfeeding of infants, from below 1 to 23 %. The exact level of coverage for each indicator is presented in Additional file 1: Table B.2-3.

#### Outcome - equity

**Fig. 7:**
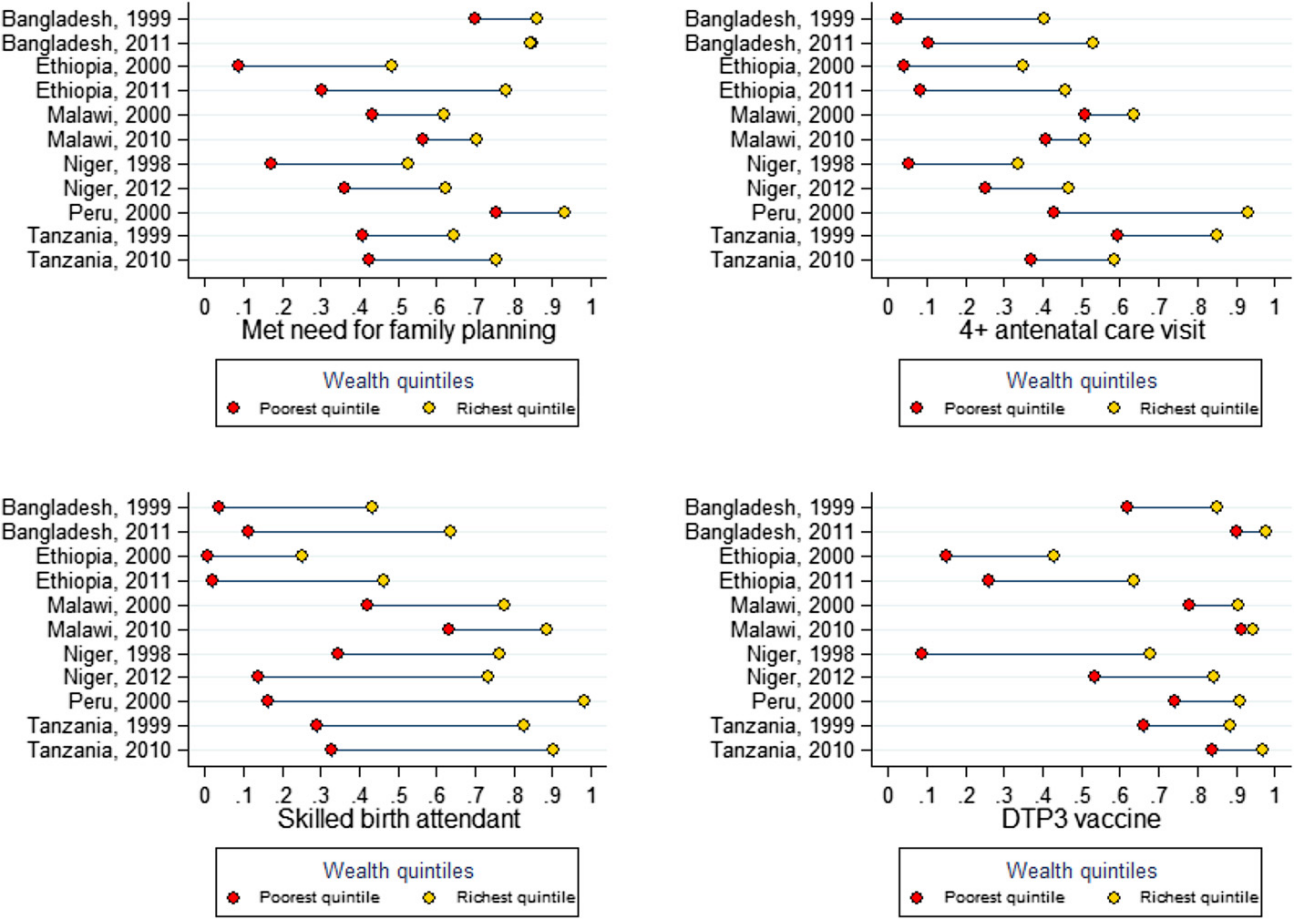
Coverage of select Commission on Information and Accountability (CoIA) indicators for Countdown to 2015 case study countries, in the poorest and richest wealth quintiles, over time (%). Figure 7 includes only those case study countries with available data. Antenatal care and skilled birth attendance are reported among births during the 3 years preceding the survey

The coverage statistics above represent all-population averages. A more nuanced story emerges when we examine how CoIA indicator coverage varied over time across socioeconomic groups. Figure 7 displays the equity gap, represented by the line that connects the coverage of each indicator for the poorest and richest groups in a country.

Among the Countdown case study countries since the year 2000, Peru made the most significant progress in closing the equity gap on all indicators studied. It decreased the difference in coverage between poorest and richest groups by 32 percentage points for four or more antenatal visits, and 33 percentage points for SBA – though its equity gaps remain among the largest among case study countries for these indicators. Contrastingly, the equity gap increased for all indicators in Ethiopia over this period, by 23 percentage points for SBA, and nearly 10 percentage points for demand satisfied for family planning, attendance at four or more antenatal visits, and DTP3 immunisation. Both the poorest and richest quintiles in Ethiopia saw increased coverage of these interventions over the period – but richer groups saw greater improvements, which caused the equity gaps to increase (see Fig. 7).

#### Assessment of contributors to mortality change

**Fig. 8:**
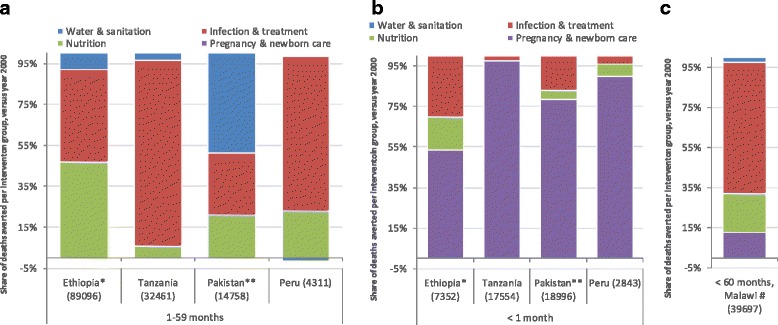
Estimated lives saved in Countdown to 2015 case study countries according to Lives Saved Tool (LIST) analyses which are associated with coverage of key interventions. **a** Children aged 1–59 months. **b** Newborns <1 month. **c** Children aged 0–59 months. All countries examine the year 2012 versus 2000 – except Ethiopia (*) which compares the year 2011 to 2000; and Pakistan (**) which compares 2012 to 2006. Negative numbers indicate a decrease in the coverage of an intervention over the period. LiST results from Malawi include averted deaths among children aged 0–59 months (#). Pregnancy and care includes obstetrics, essential newborn care, care of sick newborns and KMC. Nutrition includes breastfeeding, vitamin A supplementation, and measures to reduce wasting & stunting. Prevention and treatment of infections also includes pneumonia, malaria and diarrhoeal treatment, ITNs, vaccines and PMTCT. NB/ Deaths averted are only relating to those that can be explained by change in coverage of intervention

The case study Lives Saved Tool (LiST) analysis results suggest ways in which changes in intervention coverage may be associated with reductions in childhood mortality. Figure 8 displays the results for LiST analyses conducted in five of the case study countries (Ethiopia, Malawi, Tanzania, Pakistan and Peru).

Increased coverage of two important interventions for preventing childhood infections – vaccines and insecticide treated nets (ITNs, to prevent malaria transmission from mosquitoes) – was estimated to be associated with many averted child deaths in the case study countries. When only changes in mortality after the neonatal period was examined (Fig. 8a), increased coverage of vaccines and ITNs were estimated to be associated with 31 % of the lesser deaths in 2011 versus 2000 in Ethiopia, and 28 % of the fewer post-neonatal and childhood deaths in Pakistan for the year 2012 versus 2006 – and as high as 64 % in Tanzania, and 72 % in Peru, for 2012 versus 2000. These gains in Pakistan and Peru were all due to vaccines, since there is low malaria transmission in these settings. Additionally, increased treatment of infections was associated with approximately 10 % of the averted post-neonatal childhood deaths over this period in Ethiopia and Tanzania, but only 2 and 4 % in Pakistan and Peru, respectively.

Nutrition improvements, i.e., reduced stunting and wasting, increased coverage of vitamin A supplementation, and improved breastfeeding, were estimated to be associated with 47 % of the decline in post-neonatal child mortality in Ethiopia in 2011 versus 2000, and 24 % in Peru for 2012 versus 2000 (Fig. 8a).

Among neonatal deaths (Fig. 8b), increased clean birth practices, labour and delivery management, and postnatal care for all neonates and thermal and kangaroo mother care, were estimated to be associated with 35 % of the fewer neonatal deaths in Peru in 2012 versus 2000, 33 % of the reductions in Tanzania over this period, and 44 % in Pakistan for 2012 versus 2006.

#### Assessment of changes in social and economic factors

The socioeconomic and development context in the case study countries changed substantially between 1990 and 2013 (detailed information in Additional file 1: Table B.2-4), and change was heterogeneous across the case study countries. For example, Ethiopia saw large improvements in access to clean water and improved sanitation, with the most substantial gains occurring during the 1990s: between 1990 and 1999, access to improved sanitation increased by 13.3 % per year and by 7.5 % between 2000 and 2013, and access to safe water increased by 7.8 % per year during 1990–1999 as compared to 4.1 % for the 2000–2013 period. Peru began the MDG era with relatively high levels across the key socioeconomic factors analysed, such as per-capita gross national income (approximately quadruple the average value from the other case study countries, at over 3000 USD in 1990), female literacy (80 % and above throughout this period), urbanisation (69 % of the population in 1990), and access to safe water and sanitation (75 and 54 % in 1990, respectively).

### Objective 2: Undertake content analysis research to explore factors that may have enabled or hindered progress towards achieving MDGs 4 and 5

#### Governance and leadership

Several case studies highlighted examples of how political commitment and strong leadership aided progress towards MDGs 4 and 5. For example, Tanzania has had strong and consistent political stability for decades and has seen a recent proliferation of RMNCH policies resulting in the development of an integrated and comprehensive “One Plan” [11]. Peru’s leaders demonstrated strong and continued political commitment to improving the health of mothers and children throughout the MDG era, allowing Peru to sustain macro policies despite changes in leadership, resulting in long-term pro-poor health policies for RMNCH [10]. Similarly, Ethiopia’s government adopted and backed a comprehensive 20-year health sector strategic plan in the 1990s, including the introduction of its Health Extension Programme which has been singled out as a successful step for improving healthcare delivery at the community level [7]. Malawi’s government also demonstrated strong formal leadership through the early adoption of evidence-based policies for child survival [8]. Afghanistan signed the Millennium Declaration in 2004, a demonstration of political commitment, despite ongoing instability [6]. In contrast, Pakistan has yet to see sustained political commitment or support for maternal and child health [9].

A lack of political commitment was cited by the case study teams as a key factor in explaining the relative inattention to newborn health until more recently, compared to post-neonatal child health. Newborn health was not on the global agenda until the early 2000s when the large and growing percentage of child deaths occurring in the neonatal period, and the preventable causes of neonatal deaths were highlighted in two Lancet series [23, 24]. Increased awareness of the evidence led to increased political attention, and many of the case study countries introduced policies and programmes specifically targeted to newborns; for example, a National Child Survival Strategy was introduced in Ethiopia in 2005 to address neonatal and child mortality [7], and Tanzania conducted a Situational Analysis in 2009 to specifically introduce strategies for reducing newborn mortality [11]. Malawi’s attention to newborn survival also intensified after 2005 with a new roadmap to reduce maternal and neonatal mortality [8], and the Peru case study specifically discussed how the Lancet series informed its newborn policies, which now include national scale-up of neonatal care [10].

Health system governance structures were also cited as affecting RMNCH policy and programme adoption. Three case study countries introduced decentralisation to improve intervention coverage for all population groups. The case studies from Peru and Ethiopia mentioned how decentralisation has increased the active participation of local and regional governments in the design and implementation of RMNCH programmes [7, 10], while in Tanzania, decentralisation has provided financial resources for health programmes to districts since 2000 [11]. However, health system decentralisation can have mixed results, as in Peru where the Ministry of Health saw reduced capacity to perform its functions outside of the capital city [25].

#### Health system financing

Most case study countries have seen increased financial flows to RMNCH, with the exception of Pakistan, where the total expenditure on health (as a percentage of the gross domestic product) has remained stagnant [19]. However, overall expenditures remain low in relation to international benchmarks for almost all the Countdown case study countries [26, 27], and this is especially true for resources mobilised domestically.

Peru, a middle-income country, experienced considerable economic growth over the MDG period, which translated into more resources available for effective intervention implementation and scale-up across the CoC for RMNCH [10]. The other case study countries are lower-income countries and have been more dependent upon external funding sources (Malawi, Tanzania, Ethiopia, and Afghanistan), which overall increased over the study period [28].

Health financing across the CoC is variable among the case study countries in terms of both level and functions supported; findings on these trends in financing are discussed in detail elsewhere in this supplement [28]. Maternal and neonatal health have generally received less funding than child health, and several case studies – including Afghanistan, Ethiopia, Malawi and Tanzania – attributed this to donors’ emphasis on vertical and disease-specific programmes: high impact interventions for child health (e.g., immunisation, ITNs and Integrated Management of Childhood Illness (IMCI)) have received substantial external financing [6–8, 11].

#### Health workforce

Four case study countries cited shortages in skilled human resources, including inequitable geographic distribution of available health workers, as a major bottleneck to MDG progress (Ethiopia, Malawi, Pakistan, Tanzania). The poorest areas of Afghanistan have seen the smallest growth in health worker cadres, and a multivariable analysis found that low nearby availability of midwives was associated with lower likelihood of skilled birth attendance and of facility birth [11]. There have been some innovative approaches to expand the numbers and roles of lower level workers. For example, Ethiopia developed the Health Extension Programme to address increasing demand for primary health care [7], and Malawi introduced an emergency human resources plan, which increased the number of health care workers by 53 % between 2004 and 2010, including a more than two-fold increase in Health Surveillance Assistants [8] – although both case studies mentioned concerns about quality of health workers.

#### Medicines and commodities

Stock-outs of medicines and supplies were commonly mentioned in the case studies as hindering delivery of high quality, effective services. For example, Pakistan reported that only 37 % of basic health units have all critical medicines in stock, including modern contraceptive methods [19]; and the Tanzania case study found geographic disparities in stock-outs of family planning commodities [11]. Several case study countries have worked to increase access to medicines and commodities. Strengthening pharmaceutical and medical supply availability is a priority area in Ethiopia’s Health Sector Development Programme [7]; and Malawi established a Central Medical Stores Trust to improve the pharmaceutical supply chain, although stock-outs are reportedly still common [8].

#### Health service delivery, quality, and utilisation

Several case studies noted a shift after the year 2000 in programme focus, from high impact “vertical” interventions, such as the Expanded Programme on Immunisation (EPI) to the introduction of more integrated approaches to RMNCH services such as IMCI, ICCM and other community-based health programs. An example of the latter is Afghanistan’s community-based health care and community midwife programmes, to which the case study attributes Afghanistan’s rapid recent increases in SBA and antenatal care [6]. Similarly the HEP and construction of health posts were cited by the Ethiopia case study as associated with remarkable gains in primary health care coverage [7].

Additionally, Peru has introduced health reform initiatives, with a targeted multi-sectoral approach and a focus on women and children in poor areas – and the case study from Peru found that this was associated with improved access and utilisation of health services, such as antenatal care and skilled birth attendance, as well as a reduced equity gap between the rich and the poor and between urban and rural areas [10].

Several case studies illustrate that policy adoption alone is insufficient if not followed by effective policy and programme implementation. For example, Tanzania developed a “One Plan” to consolidate the fast-growing landscape of domestic policies and strategies around RMNCH – but it lacked a clear operational structure and costing system, and therefore was never fully implemented [11, 29]. Case studies from Ethiopia, Malawi and Tanzania noted that such implementation failures were found to be particularly evident for maternal and newborn health, as these programmes which may require more complex implementation than child health interventions, often received less consistent and effective execution, despite strong political commitment [7, 8, 11].

Poor health service quality was often cited as a barrier to progress, particularly for maternal and newborn health. As discussed above, although several countries have expanded their health workforce (e.g., Malawi, Ethiopia, and Afghanistan), these health workers may lack the skills to manage complicated conditions [6–8]. Likewise, case studies from Ethiopia and Peru discussed how the number of health facilities has increased, but poor quality may hinder outcome improvements [7, 10].

#### Country context

One common contextual factor driving progress discussed in the case studies was general economic growth leading to poverty reduction. All of the case study countries saw economic growth over this period, which has contributed to development, but widespread poverty and limited resources persist in many countries.

Political stability was also discussed by the case study teams as central to MDG progress. Tanzania experienced decades of political stability, permitting its government to promote the MDGs [11]. On the other hand, Afghanistan has long experienced insecurity and conflict, but has achieved progress in the past decade, particularly enabled by external donor support and the reconstruction process [6]. Ethiopia, which had previously been affected by widespread and protracted conflicts, has seen greater stability under the new government established in 1991; its new constitution (ratified in 1994) prioritises principles of democracy and equity, and created a paradigm shift in governance and policy-making including in health [7]. Peru’s transition to democracy during the early 2000s encouraged broad participation in policy decision-making, including active participation of civil society – which the case study described as instrumental to fostering political will and commitment to RMNCH at all levels, and may have facilitated Peru’s outstanding performance on the MDGs despite some political and economic unrest before this period [10].

Country teams such as Ethiopia and Malawi drew linkages between gains in child health and improvements in food availability and consumption. Against its history of conflicts, poverty and food crises, for example, Ethiopia made substantial efforts to address nutrition, agricultural productivity, and to introduce a safety net against droughts [7]. In Malawi, coverage gains were achieved in community-based nutrition interventions in the mid-2000s and the country experienced an increase in food security [8].

## Discussion

The Countdown country case studies used mixed methods to comprehensively assess why and how selected countries in Asia, Latin America and sub-Saharan Africa achieved or failed to meet MDGs 4 and 5. A standard framework and methodology were applied for each case study country, which enabled us to systematically review findings and identify key lessons learned.

Seven of the 10 case study countries (China, Peru, Malawi, Bangladesh, Ethiopia, Niger and Tanzania) achieved MDG 4 to reduce childhood mortality, with particular gains seen after the year 2000, and in deaths between the ages of 1 and 59 months. Neonatal mortality gained attention from the mid-2000s, and improved more slowly. There was also progress, although to a slightly lesser degree, toward reducing maternal mortality (MDG 5a), particularly in Afghanistan, Bangladesh, China and Ethiopia. These findings are similar to those reported for the full Countdown project [1] and elsewhere in the literature [6, 8].

### Success in scaling up interventions delivered in communities and via primary health care system

The case study countries were most successful in scaling up interventions that can be delivered via the primary health care system and in communities, especially child health interventions. There has been widespread implementation of high-impact interventions that can be administered via low levels of the health system, especially for child health. A prime example of this is childhood vaccinations, which began decades ago with the EPI programme, now has broad global support, including substantial financing, and a robust evidence base [30, 31], and includes newer vaccines for pneumococcal disease, rotavirus, and *Haemophilus influenzae type B* (Hib). The LiST analyses suggest that the scale-up of immunisations, especially these newer vaccines, may have been associated with many of the post-neonatal infant and childhood lives saved in the case study countries by the year 2012.

The Countdown case studies suggest mechanisms for improving equity, such as in Peru, where the country’s pro-poor and targeted implementation strategy of childhood immunisation increased equity in health outcomes. The Pakistan case study highlighted the potential riskiness of tightly focusing on a single intervention: the recent emphasis on polio eradication was cited as a detriment to uptake of routine childhood immunisations [19].

The case study countries’ initiatives to strengthen lower levels of the health system, including community-based programmes and strengthening cadres of lower-level health workers, were also seen as important for improving access to key interventions. For example, although not specifically analysed here, the Niger case study discussed how investments into universal primary health care, including health system strengthening and introducing a new cadre of community health workers, were key factors in the country’s improvement in child survival [4].

Although there is robust evidence on the importance of meeting the need for family planning [32, 33], which can often be achieved via the primary health care system or at the community level, this was generally not explored in the case studies – with the exception of the Tanzania case study, which noted the highly variable commitment to, and implementation of, reproductive health programmes since the 1980s [11].

### Variable and lesser increases in coverage of interventions that must be delivered through middle- and higher-level facilities

Interventions delivered via higher-tiered facilities as part of a functioning health system also have persisting equity gaps and have obtained less external support. A key example of this is SBA, which had the most variable level of coverage among all the indicators, as well as the largest equity gaps, which echoes global Countdown findings [1].

The case studies showed that supportive programmes and policies arrived later for maternal and neonatal interventions, corresponding to a global delay in attention for these issues [34]. This may reflect the lack of clear, universally agreed-upon strategies for maternal and neonatal care, despite repeated proposals and an evidence base on effective interventions [35–40]. This lack of a global consensus saw a parallel in the country case studies – which, for example, reported a variety of approaches to improving neonatal health, from scaling up neonatal resuscitation and Kangaroo Mother Care in Tanzania, to implementing a community-based newborn care model in Ethiopia and Malawi.

The case study results are also consistent with the evidence base showing that there may be a limit to how much a country’s U5MR may be lowered if its NMR remains high. Continued improvements in childhood survival may also require more complex interventions, e.g., treatment coverage for pneumonia and diarrhoeal disease as well as improved nutrition programmes, and these lagged over the study period in case study countries. [1]. The global health community must emphasise further improvements in these areas.

Health system constraints, including health worker shortages, were identified by the case study countries as a major barrier to achieving the MDGs, as has been reported elsewhere in the literature [41], particularly for decreasing maternal and neonatal mortality [13, 42, 43]. Although several of the case studies cited poor quality of care as a potential limiting factor to progress [44–47], this is difficult to quantify and was not explored empirically in these case studies. This is an area where future research is clearly needed.

Governments, in partnership with the donor community, must continue focusing on health system strengthening. The Sustainable Development Goals’ framework promotes the achievement of universal health care, which requires emphasis on improving health systems, particularly in countries faced with an unfinished agenda of high maternal and child mortality coupled with increases in non-communicable diseases.

### Political, economic and social factors contributed to MDG progress

Political, social and economic factors enabled progress in RMNCH, though the extent is difficult to assess and quantify. These findings are consistent with the emphasis on multi-sectoral approaches to addressing mortality and development in the new Sustainable Development Goals [48].

It is clear from the literature that social and economic development, as well as political context and shifts across non-health sectors, influenced health outcomes and affected the implementation of health policies and programmes including the MDGs [49–51]. The case study countries experienced many political, economic and social changes during the MDG era, and nearly all the case studies discussed the importance of context in influencing progress. The Bangladesh case study, although not included in this comparative content analysis, also showed both health and non-health sector factors (such as improvements in household wealth and women’s education) were crucially important in explaining reductions in maternal mortality [5].

The case studies indicate that changes in both context and coverage contribute to improved maternal, newborn and child survival. However, further research is needed to precisely attribute their relative contributions in each setting. For example, the case study LiST analyses did not entirely predict the actual survival improvements seen since 2000: it accounted for 80 % of the reported mortality reduction in Malawi over this period, 73 % in Peru, 51 % in Ethiopia, and 39 % in Tanzania. This may be because LiST analyses do not capture all coverage-related changes that may be relevant for mortality decline. They also do not model the role of non-intervention factors, such as infrastructure, economic development, or changes in social and demographic determinants such as education [52, 53] – which are likely also important for mortality reduction.

An important example of the complex interplay of policies and context with health outcomes is childhood nutritional status. Several case study countries with good progress on stunting took multi-sectoral approaches to addressing this problem, such as introducing reforms in the agricultural sector coupled with nutrition-specific programmes delivered through the health sector, as well as large gains in the coverage of improved water and sanitation. The Niger case study also hypothesised that its multipronged approach to addressing under-nutrition – including both ongoing and emergency services – was an important factor in reducing childhood mortality [4]. Effective mechanisms to reduce under-nutrition are crucially important, as it is estimated that this is the cause of 45 % of all deaths among children under age 5 [54].

### Measurement is key for effective implementation and monitoring of global initiatives

These results underscore the importance of measurement for the effective implementation and monitoring of global initiatives like the MDGs and now, going forward, the Sustainable Development Goals.

The Countdown initiative, including the case study analyses, have emphasised the centrality of high-quality data for evaluating progress, including identifying lessons learned and remaining gaps, and for programme monitoring purposes. Although Countdown and others have helped generate momentum for improved measurement and accountability globally and in some countries, there is still a long way to go. Data collection has increased for many indicators [1], but remains a challenge in many countries, particularly those that lack vital registration systems; only one case study country (Peru) contributed vital registry data to the latest WHO maternal mortality estimate. Although some interventions may be comparatively well-monitored, such as immunisations (for which data systems often exist to track antigen-specific coverage at a sub-national level, as well as information on vaccine financing, supply chain issues such as stock-outs, etc.), other domains are less well understood. An important example is neonatal mortality, which is inconsistently defined and measured across settings (including how intrapartum stillbirths are classified, as well as when and how births are recorded), when it is measured at all.

Reliable, frequent and timely data on the coverage and equity of interventions, and on health outcomes, can inform policy adoption and implementation of programmes. Regional- or district-level information can be used to guide implementation, and to measure progress [55]. Such data can also be used to inform research endeavours, such as the LiST analyses included here, which themselves can inform policy-making and priority-setting. Additionally, mixed methods research approaches, like the Countdown case studies, are an important means for going beyond summary indicators, and assessing the “how” and “why” of progress (or not). Such studies should continue to be undertaken during the Sustainable Development Goals era.

Information availability and data democratisation can enable the engagement of a wide range of stakeholders in discussions about health policies and programmes. The case studies demonstrate that data collection, analysis and synthesis are only the first steps in promoting data use by decision-makers and advocates for action. Some of the case study countries, such as Tanzania, engaged local stakeholders in discussions about the data and progress toward MDGs 4 and 5, which helped stimulate local ownership of the results. More work is needed on refining the Countdown case study model so that dissemination efforts lead to greater uptake of findings for programming and planning purposes. Additionally, more work remains to be done on how to incorporate the private sector into case study analyses, to develop a fuller picture of factors driving progress.

Recent discussions have emphasised the importance of data in sustained global and national accountability in achieving future gains in RMNCH, in health more broadly, and in the context of achieving universal health coverage [56–58].

The Countdown case study portfolio has several strengths, including geographic representation from countries in Africa, Asia and South America; a standard evaluation framework, with a mixed methods analysis approach to attempt to capture the complex factors driving progress; and national capacity-building and engagement of a range of stakeholders in the case study process. This last point in particular distinguishes the Countdown case study process as offering a unique, locally-driven perspective on important RMNCH topics.

There were a number of limitations to our study that should be noted. First, there were a small number of case studies conducted and there may have been case selection bias, since the countries were selected on a number of factors that may be correlated with degrees of change, including data availability and in-country capacity to undertake the research. Secondly since the case studies were largely focused on post-neonatal child health outcomes, the content analysis presented in this cross-cutting paper could not robustly examine reproductive, maternal, neonatal, nor adolescent health. This underscores the importance of increasing attention to these parts of the continuum of care, and perhaps more thoroughly integrating research and practice regarding the more neglected areas of reproductive, adolescent, and neonatal health with the generally more successful maternal and child health issues. We were therefore limited in our ability to explore certain hypotheses – particularly since the case study countries were not selected as representative of certain outcomes (as has been done in other research, such as the Success Factors case studies [59]) so formed a non-systematically heterogeneous dataset. Thirdly, the content analyses were limited to materials made available from the second phase country teams. We aimed to minimise biases by having two investigators separately conduct the content analysis and by vetting our conclusions with the case study team members. However, we note that there were limited data for health information systems, and evaluation of political, governance and leadership aspects, which are crucial to understanding progress and require further analyses [55]. Lastly, the measurement of many of the quantitative indicators – including mortality outcomes, which are modelled estimates usually based on household-level nationally representative surveys, as well as coverage and equity data, which are based on nationally representative DHS surveys – face limitations such as small sample size and challenges with estimation procedures; implications of this for LiST analysis results are discussed at length elsewhere [53].

## Conclusions

The Countdown case studies present a complex picture of progress toward MDGs 4 and 5 (Table 1). The results indicate that achieving mortality change at a population level is not merely a technical process of adopting policies or administering programmes. Rather, an interchange of factors which determines intervention coverage and equity, which subsequently affect health outcomes and impact.

**Table 1:** Key messages

***Key messages***
1. **MDG progress especially for child survival:** seven of the 10 Countdown case study countries met Millennium Development Goal (MDG)-4 to reduce their under-5 mortality rate by two-thirds between 1990 and 2015. Key childhood interventions (e.g., immunisations and insecticide treated nets in malaria endemic countries) saw major increases in coverage, partly due to their delivery at community and primary health care level, as well as to global and national commitment to these interventions, which manifested in greater financial resources and focused attention on implementation.
2. **Slower progress for neonatal and maternal mortality:** these reductions were generally more modest, though newborn health did not receive attention until the mid-2000s. There was slower progress in the coverage of intrapartum interventions such as skilled birth attendance, with persisting large equity gaps. The case studies reported lower political commitment, less financing, and more health system constraints for implementing intrapartum interventions, partly because they must be delivered through middle- and higher-level facilities as part of a functioning health system.
3. **Reproductive health:** few case studies explored progress in improving reproductive health. Fertility levels did not reduce dramatically over the MDG era in most of the countries, and family planning received comparatively lesser funding than did child health, and particularly HIV/AIDS, although investments have increased since 2010.
4. **Systematic methods to compare country progress:** this portfolio of case studies demonstrates how mixed methods research can provide insights into the “how and why” of improving women’s and children’s health, by using a standard evaluation framework and engaging multidisciplinary, independent country teams in collecting and analysing data from a variety of sources. More data and further research advances are needed, including better understanding of the role of social, economic and political factors, including leadership and governance.
5. **Future of women’s and children’s health:** as the world transitions into the Sustainable Development Goals era, continued investment is crucial for the unfinished agenda of improving maternal, child and newborn survival, as well as for ensuring they thrive and transform into productive citizens. Improved data are required especially at subnational level and to drive these improvements in coverage and equity, but also quality, so no women or their children are left behind.

This comparison across the case study countries, as well as findings from the Countdown report looking across all 75 priority countries [1], indicate that substantial progress was achieved during the MDG era in the highest burden countries – particularly for interventions that had greater global buy-in and financing. With more focus (attention and funding) came greater coverage and equity, which suggests a clear future path forward: we must continue to implement these approaches that have so far been successful and also must increase attention to other interventions and to underlying health system strengthening, including hospital care for sick newborns, for more effective, equitable and efficient policy and programme implementation. Improvements in sectors outside of health such as in agriculture and education are also critical.

The case studies were developed by country-based researchers and policymakers, supported by the Countdown technical working groups. Many case studies had strong local buy-in and some have resulted in real change at the national level. An example of this can be seen in Tanzania, where the Countdown case study results were launched by the President and used to develop the Sharpened One Plan, which aimed to accelerate reductions in the NMR, U5MR and MMR [11]. This reflects an important shift towards a research model that emphasises capacity building and engagement of stakeholders at the country level. Such exercises need to be replicated, and improved measurement should be a key priority in future efforts.

Data show that maternal and child mortality reduction accelerated over the last five years. We are at a critical juncture as the world moves into the Sustainable Development Goals era, and must commit to doing more – not less to finish the unfinished agenda of RMNCH. The Countdown case studies demonstrated that focused attention, financing and effective implementation can make a real impact on improving equitable intervention coverage and saving lives. They also underscore the importance of progress monitoring, which depends on the availability of reliable data, as essential for accountability. In the years ahead, the global community must sufficiently invest in data systems and other health system strengthening efforts to improve accessibility of high quality care while also addressing the underlying social and political determinants of health. We also must gain an improved understanding of how to improve outcomes for special populations – including adolescents, and groups affected by conflict and other humanitarian emergencies; these were largely not explored by the Countdown case studies but should be critical priorities over the coming decades. Compared to the MDG era, an even greater level of ambition, investment, measurement and accountability is required to address the “survive, thrive and transform” agenda in the new Global Strategy for Women’s, Children’s and Adolescents’ Health [60].

## Abbreviations

CoC, continuum of care; CoIA, United Nations Commission on Information and Accountability for Women’s and Children’s Health; Countdown, countdown to 2015; DTP3, diphtheria-tetanus-pertussis immunisation; EPI, Expanded Programme on Immunisation; Hib, Haemophilus influenzae type B immunisation; ICCM, Integrated Community Case Management; IMCI, Integrated Management of Childhood Illness; ITNs, insecticide treated nets; LiST, Lives Saved Tool; MDG, Millennium Development Goal; MMR, maternal mortality ratio; NMR, neonatal mortality rate; RMNCH, reproductive, maternal, newborn and child health; SBA, skilled birth attendance; U5MR, under-5 mortality rate; WHO, World Health Organisation

## Additional file

Additional file 1: Additional material. (DOCX 112 kb)

## Footnotes

1 Although stillbirths are an important impact measure, they were generally not investigated by the case study teams, so are not discussed in this paper.

2 The Pakistan analysis was still underway at the time of this publication so only minimal information could be included; similarly, China and Kenya were not included because their case study analyses were incomplete at the time of publication.
