# Supplementary material for: Countdown to 2015 country case studies: what have we learned about processes and progress towards MDGs 4 and 5?
Source: BMC Public Health. 2016 Sep 12;16(Suppl 2):794. doi: 10.1186/s12889-016-3401-6 (PMC5025828; doi:10.1186/s12889-016-3401-6)
Supplement: Additional file 1: — Additional material. (DOCX 112 kb) [file 12889_2016_3401_MOESM1_ESM.docx]

**Countdown to 2015 country case studies: What have we learned about processes and progress towards MDGs 4 and 5?**

**Additional file 1**

Contents

[A. Acronyms 2](#_Toc455050027)

[B. Supplemental methodology, results, tables and figures for Paper 1 - What have we learned about national progress towards MDGs 4 & 5? 4](#_Toc455050028)

[B.1 Countdown country case studies: Evaluation framework, and description of process 4](#_Toc455050029)

[B.2. Quantitative data analysis 9](#_Toc455050030)

[C. References 16](#_Toc455050031)

# Acronyms

AMDD Additional Death and Disability Programme

ANC Antenatal Care

CHW Community Health Worker

CoC Continuum of Care

DHS Demographic Health Surveys

EBF Exclusive Breastfeeding

EHP Essential Health Package

EHRP Emergency Human Resources Plan

EmOC Emergency Obstetric Care

GDP Gross Domestic Product

GNI Gross National Income

HEW Health Extension Workers

HSP Health Systems and Policies

ICCM Integrated Management of Newborn and Childhood Illness

IMCI Integrated Management of Childhood Illnesses

LBW Low Birth Rate

LiST Lives Saved Tool

LICs Low Income Countries

LYS Life Years Saved

MBB Marginal Budgeting for Bottlenecks

MDG Millennium Development Goals

MMR Maternal Mortality Ratio

NMR Neonatal Mortality Rate

NGO Non-Governmental Organisation

OOP Out-of-Pocket (spending)

PHC Primary Health Care

PNC Postnatal care

PPP Public Private Partnership

PSBI Possible Serious Bacterial Infection

RMNCH Reproductive, Maternal, Newborn and Child Health

RCT Randomised Control Trial

SDG Sustainable Development Goals

SRG Scientific Review Group

SWAp Sector Wide Approach

THE Total Health Expenditure

TFR Total Fertility Rate

U5MR Under-5 Mortality Ration

UN United Nations

UNCoLSC United Nations Commission for Life Saving Commodities for Women and Children

UNFPA United Nations Population Fund

UNICEF United Nations Children's Fund

USD United States Dollar

WASH Water and Sanitation

WHO World Health Organisation

# B. Supplemental methodology, results, tables and figures for Paper 1 - What have we learned about national progress towards MDGs 4 & 5?

# B.1 Countdown country case studies: Evaluation framework, and description of process

1. **The evaluation framework**

An evaluation framework used to guide each of the in-depth case studies. It presents hypothesised pathways through which key inputs and outputs, as well as broader contextual factors are expected to influence intervention coverage and equity outcomes, and ultimately population health. The key constructs are described below, as well as how they were operationalised for each case study’s research.

- *Coverage and equity:* The case study teams analysed trends in coverage of effective interventions across the RMNCH continuum of care (CoC). Since aggregate national-level coverage statistics may fail to reveal important within-country health and coverage inequalities, Countdown case study teams also explored patterns of coverage, stratified by wealth, geographic region, and urban and rural location [[1](#_ENREF_1), [2](#_ENREF_2)].
- *Health systems and policies:* To better understand coverage patterns and trends, the case study teams documented when their respective countries adopted supportive policies and introduced national RMNCH programmes, and identified any major bottlenecks countries experienced when bringing programmes to scale or delivering services to women and children at each level of the health care system. Paper 2 [[3](#_ENREF_3)]of this supplement presents the standardised HSP tools developed to help country teams capture information on the systems and policy environment.
- *Financing:* Because adequate financing is essential for effective program implementation, case study teams analysed financial flows in the health sector, and specifically to women’s and children’s health, and explored how these flows were associated with political commitment and performance. Paper 3 [[4](#_ENREF_4)] of this supplement presents the methodology used for assessing the relationship between health care financing and case study countries’ progress toward MDGs 4 and 5.
- *Lives Saved Tool (LiST):* This methodology uses data from nationally representative surveys to estimate coverage of key interventions relevant to child survival, and estimates the relative contribution of these interventions to mortality reduction (using Spectrum software). The Countdown country case study teams conducted LiST analyses, with support from the LiST Technical Working Group [[5](#_ENREF_5), [6](#_ENREF_6)]; each country chose the relevant interventions (based on their own burden of disease), time frame, and age range (i.e., for all childhood deaths, or separate analyses for neonatal and post-neonatal childhood deaths).

1. **Case study process overview**

Countdown case study selection: The first two case studies were carried out in Niger and Bangladesh in response to Countdown’s recognition of the need to complement the initiative’s global-level monitoring with in-depth analyses of the “how and why” of progress within countries. Niger and Bangladesh were selected on the basis of data availability, an important story to tell about progress reducing child mortality and maternal mortality respectively, and existing strong collaborative partnerships between academic institutions involved in Countdown and research institutions in these two countries. These two studies generated substantial interest from other countries for similar analyses, which led to Countdown developing a full portfolio of case studies, selected as described below.

From the 75 Countdown countries, nine were initially selected to submit proposals based on data availability, progress toward the MDGs, and non-duplication with other Countdown case studies. The proposals were then ranked, using the Child Health and Nutrition Research Initiative (CHNRI) systematic scoring procedure [[7](#_ENREF_7)], by seven Countdown Scientific Review Group (SRG) members, and representatives from USAID and UNFPA. The top scorers then submitted more detailed proposals outlining the country situation, proposed case study activities and budgets. Six country case study teams were ultimately selected by the SRG in February 2013. Early in 2014, an additional nine countries submitted proposals, from which two (“round 2”) case study teams were selected.

Data sources and methodology: An overview of the steps involved in each case study is presented in Figure B.1-1. The objectives, data sources and analysis methods used within each domain of the Countdown country case studies are outlined in Table B.1-1. High-quality, reliable and relevant data across the components of the evaluation framework (manuscript Figure 1) were collected and analysed, as well as data regarding programme and policy investments, and breadth and strength of implementation.

The Countdown case studies were led by institutions in each country that are independent of RMNCH programme implementation. All analysis plans and preliminary results were presented and discussed with policymakers, government and other relevant stakeholders. Country workshops to strengthen capacity in data collection and analysis were organised, with the collaboration of academic institutions, statistical offices, the MoH, local partners and the global Countdown team. Each case study team also conducted a dissemination event at the conclusion of the process.

Each Countdown case study assessed at least one impact endpoint, and related outcomes along the RMNCH CoC, over time. There has been sustained global attention around child health – and all of the Countdown country case studies (except Bangladesh) identified child health as a priority. Only two case studies (Bangladesh and Tanzania) discussed reproductive health.

**Table B.1-1. Objectives, data sources and analysis methods used within each domain of the Countdown to 2015 country case studies**

| Domain | Objective | Data Sources | Analysis Methods |
| --- | --- | --- | --- |
| **HEALTH**  **SYSTEMS &**  **POLICIES** | *To track selected health systems and policy indicators necessary to increase access to and utilisation of RMNCH interventions, and to assess strength of RMNCH policy to programme implementation* | 1. MNCAH Survey (WHO) 2. UNCOLSC 3. UNFPA 4. AMDD 5. National and international policy documents | - Health Policy Tracer Indicators Dashboard - Health System Tracer Indicators Tool - Policy & Programme Timeline Tool - Approaches to assess implementation strength |
| **FINANCING** | *To track and analyse financial flows* | 1. OECD’s Creditor Reporting System (CRS) 2. Official Development Assistance (ODA) 3. National Health Accounts 4. Public Expenditure Tracking Surveys | - Health Resource Tracking (HRT) - Analysis of financial protection and access to services - Analysis of equity in public financing of health programmes - Costing |
| **COVERAGE** | *To ensure that measures of coverage reflect the best available indicators and data, reported in standard and transparent ways across countries and over time* | 1. Demographic Health Surveys (DHS) 2. Multiple Indicator Cluster Surveys (MICS) | - Composite coverage index [[8](#_ENREF_8)] - Co-coverage indicator |
| **EQUITY** | *To carry out analyses of survey results and provide a breakdown of key RMNCH indicators according to the different dimensions of equity* | 1. DHS 2. MICS | Socio-Economic Positions (SEP)   - No single measure:   - Absolute/relative evaluations complementary |

**Figure B.1-1. What is involved in undertaking an in-depth Countdown to 2015 country case study?**

**Figure B.1-2. Countdown to 2015 case study country selection process**

# B.2. Quantitative data analysis

1. **Details on data sources and analysis**

This cross-cutting analysis examined impacts: total fertility rate (TFR), neonatal mortality rate (NMR), under-5 mortality rate (U5MR), and the maternal mortality ratio (MMR) [[9-12](#_ENREF_9)]. This paper also uses data from the United Nations Commission on Information and Accountability (CoIA) for Women's and Children's Health [[13](#_ENREF_13)] on: demand for family planning satisfied (among married women), attendance at 4 or more antenatal visits (among women with births during the prior 3 years), skilled birth attendance (among women with live births during the prior 3 years), exclusive breastfeeding for infants (aged 0-5 months), diphtheria/tetanus/pertussis vaccine (DTP; all three doses, among children age 12-23 months), and prevalence of stunting among children. These data are accessible at [www.countdown2015mnch.org](http://www.countdown2015mnch.org).

Changes per year for impact and coverage indicators – i.e., annual average rates of change – were calculated using the standard formula: [(Value in year 2 / Value in year 1) ^ ((Year 2-Year1^-1))-1]. For coverage time trend analyses, only countries with at least two data points per indicator since the year 2000 were included, so this represents both a subset of the case study countries and a subset of the CoIA indicators.

Additionally, results from the countries’ own LiST analyses, which link across levels of the evaluation framework, were used to assess the contribution of intervention coverage to mortality change. LiST results were obtained directly from the country case study teams; further detail pertaining to their data sources and methodology can be found in each case study’s main paper [[14-18](#_ENREF_14)]. For this cross-cutting analysis, the case study LiST results were aligned by time period (the year 2012 versus 2000, in all cases except Ethiopia which compared the year 2011 to 2000, and Pakistan which examined 2012 versus 2006) and age group (children aged 0-59 months), and the interventions were standardised so they could be compared across countries.

The social and economic indicators investigated in this cross-cutting paper are those utilised by the Maternal and Child Epidemiology Estimation group (omitting those that overlap with coverage, outcome or impact indicators otherwise investigated by the case study teams) (MCEE). The human development index, urbanisation, female literacy and education attainment (years of schooling) data were from the United Nations Development Programme; data on coverage of water and sanitation (WASH) came from the WHO/UNICEF Joint Monitoring Programme for Water Supply and Sanitation; the Gini coefficient and the gross national income (GNI) data are from the World Bank. All data were obtained from MCEE [[19](#_ENREF_19)]. Data were collated in a database, using standardised definitions and units; data points were smoothed using linear interpolation to account for missing years.

Table B.2-1 shows the representation of each case study country across the methodologies used for this cross-cutting analysis.

**Table B.2-1: Countries’ representation in each component of quantitative analysis presented in this cross-cutting paper**

|  | Impact indicators | Coverage time trend | Equity time trend | LiST analysis | Contextual variables | Qualitative analysis |
| --- | --- | --- | --- | --- | --- | --- |
| Afghanistan | ✓ | ✓ |  |  | ✓ | ✓ |
| China | ✓ |  |  |  | ✓ | ✓ |
| Ethiopia | ✓ | ✓ | ✓ | ✓ | ✓ | ✓ |
| Kenya | ✓ | ✓ |  |  | ✓ | ✓ |
| Malawi | ✓ | ✓ | ✓ | ✓ | ✓ | ✓ |
| Pakistan | ✓ |  |  | ✓ | ✓ | ✓ |
| Peru | ✓ | ✓ | ✓ | ✓ | ✓ | ✓ |
| Tanzania | ✓ | ✓ | ✓ | ✓ | ✓ | ✓ |
| Niger | ✓ | ✓ | ✓ |  | ✓ |  |
| Bangladesh | ✓ | ✓ | ✓ |  | ✓ |  |

1. **Additional results**

**Table B.2-2: Levels in 1990, 2000 and 2013, and annual rates of change (ARC) over the full MDG era (1990-2015) as well as each decade within (1990-2000 and 2000-2015), for impact and outcome variables, Countdown to 2015 case study countries**

|  | China | Peru | Malawi | Bangladesh | Ethiopia | Niger | Tanzania | Kenya | Afghanistan | Pakistan |
| --- | --- | --- | --- | --- | --- | --- | --- | --- | --- | --- |
| **Neonatal mortality (< 1 month, deaths per 1000 live births)** | | | | | | | | | | |
| 1990 level | 30 | 28 | 49 | 63 | 61 | 55 | 40 | 27 | 53 | 64 |
| 2000 level | 19 | 16 | 40 | 41 | 46 | 42 | 37 | 33 | 44 | 50 |
| 2015 level | 6 | 8 | 22 | 23 | 28 | 27 | 19 | 22 | 36 | 46 |
| *1990-2015 ARC* | *-6.2* | *-4.9* | *-3.2* | *-4.0* | *-3.1* | *-2.8* | *-2.9* | *-0.8* | *-1.5* | *-1.3* |
| *1990-2000 ARC* | *-4.4* | *-5.1* | *-1.9* | *-4.0* | *-2.8* | *-2.5* | *-0.9* | *1.8* | *-1.7* | *-2.4* |
| *2000-2015 ARC* | *-7.6* | *-4.7* | *-4.1* | *-3.9* | *-3.3* | *-3.0* | *-4.4* | *-2.7* | *-1.4* | *-0.5* |
| **Under-5 mortality (< 60 months, deaths per 1000 live births)** | | | | | | | | | | |
| 1990 level | 54 | 80 | 242 | 144 | 205 | 328 | 165 | 102 | 181 | 139 |
| 2000 level | 37 | 39 | 174 | 88 | 145 | 227 | 131 | 108 | 137 | 112 |
| 2015 level | 11 | 17 | 64 | 38 | 59 | 96 | 49 | 49 | 91 | 81 |
| *1990-2015 ARC* | *-6.2* | *-6.1* | *-5.2* | *-5.2* | *-4.9* | *-4.8* | *-4.7* | *-2.9* | *-2.7* | *-2.1* |
| *1990-2000 ARC* | *-3.7* | *-6.9* | *-3.2* | *-4.8* | *-3.4* | *-3.6* | *-2.3* | *0.6* | *-2.7* | *-2.1* |
| *2000-2015 ARC* | *-7.8* | *-5.5* | *-6.5* | *-5.4* | *-5.8* | *-5.6* | *-6.3* | *-5.1* | *-2.7* | *-2.1* |
| **Age 1-59 month mortality (deaths per 1000 live births)** | | | | | | | | | | |
| 1990 level | 23 | 51 | 191 | 80 | 140 | 273 | 124 | 74 | 126 | 71 |
| 2000 level | 16 | 23 | 138 | 45 | 93 | 177 | 97 | 76 | 88 | 51 |
| 2015 level | 5 | 9 | 42 | 14 | 31 | 66 | 29 | 27 | 57 | 25 |
| *1990-2015 ARC* | *-5.9* | *-7.1* | *-6.1* | *-6.9* | *-6.1* | *-5.7* | *-5.8* | *-4.1* | *-3.2* | *-2.9* |
| *1990-2000 ARC* | *-3.3* | *-8.0* | *-3.2* | *-5.7* | *-4.1* | *-4.4* | *-2.4* | *+0.2* | *-3.5* | *-3.4* |
| *2000-2015 ARC* | *-7.6* | *-6.6* | *-8.0* | *-7.7* | *-7.4* | *-6.6* | *-8.1* | *-7.0* | *-2.9* | *-2.5* |
| **Maternal mortality ratio (deaths per 100,000 live births)** | | | | | | | | | | |
| 1990 level | 97 | 251 | 957 | 569 | 1250 | 873 | 997 | 687 | 1340 | 431 |
| 2000 level | 58 | 140 | 890 | 399 | 897 | 794 | 842 | 759 | 1100 | 306 |
| 2015 level | 27 | 68 | 634 | 176 | 353 | 553 | 398 | 510 | 396 | 178 |
| *1990-2015 ARC* | *-5.0* | *-5.1* | *-1.6* | *-4.6* | *-4.9* | *-1.8* | *-3.6* | *-1.2* | *-4.8* | *-3.5* |
| *1990-2000 ARC* | *-5.0* | *-5.7* | *-0.7* | *-3.5* | *-3.3* | *-0.9* | *-1.7* | *1.0* | *-2.0* | *-3.4* |
| *2000-2015 ARC* | *-5.0* | *-4.7* | *-2.2* | *-5.3* | *-6.0* | *-2.4* | *-4.9* | *-2.6* | *-6.6* | *-3.5* |
| **Total fertility rate** | | | | | | | | | | |
| 1990 level | 2.8 | 4.1 | 7.3 | 5.0 | 7.4 | 7.7 | 6.4 | 6.5 | 7.5 | 6.3 |
| 2000 level | 1.5 | 3.1 | 6.4 | 3.4 | 6.8 | 7.7 | 5.8 | 5.1 | 7.7 | 5.0 |
| 2015 level | 1.6 | 2.5 | 5.3 | 2.2 | 4.6 | 7.6 | 5.2 | 4.4 | 5.1 | 3.7 |
| *1990-2015 ARC* | *-2.3* | *-2.0* | *-1.3* | *-3.2* | *-1.9* | *0.0* | *-0.8* | *-1.5* | *-1.5* | *-2.1* |
| *1990-2000 ARC* | *-6.0* | *-2.8* | *-1.3* | *-3.7* | *-0.7* | *0.1* | *-1.0* | *-2.5* | *0.2* | *-2.3* |
| *2000-2015 ARC* | *0.3* | *-1.4* | *-1.3* | *-2.8* | *-2.6* | *-0.1* | *-0.6* | *-0.9* | *-2.6* | *-1.9* |
| **Stunting prevalence** | | | | | | | | | | |
| Level | n/a | 2000: 31.0%  2013: 17.5% | 2000: 54.3%  2014: 42.4% | 1999:  41.2%  2014:  36.1% | 2000: 57.4%  2014: 40.4% | 1998: 46.5%  2012: 43.0% | 1999: 48.2%  2014: 34.7% | n/a | n/a | n/a |
| *ARC* |  | *-4.3* | *-1.7* | *-2.3* | *-2.5* | *-0.6* | *-2.2* |  |  |  |

** TFR estimates cover 5-year periods concluding in 1990, 2000 and 2015 [*[*11*](#_ENREF_11)*]. MMR, NMR and U5MR are estimates for 1990, 2000 and 2015 [*[*10*](#_ENREF_10)*,* [*20*](#_ENREF_20)*] (note, 2000 estimate of NMR is from a different source [*[*12*](#_ENREF_12)*]*

**Table B.2-3: Levels of coverage of key indicators, for two time points with available data, Countdown case study countries.**

| **Country: Years of available data points** | **Indicator Percent coverage** | |
| --- | --- | --- |
|  | **Met need for family planning** | |
|  | Time-1 | Time-2 |
| Ethiopia: 2000, 2014 | 18.7% | 58.5% |
| Kenya: 1998, 2014 | 63.2% | 75.7% |
| Malawi: 2000, 2014 | 50.8% | 75.1% |
| Niger: 1998, 2012 | 33.0% | 46.5% |
| Tanzania: 1999, 2010 | 53.7% | 57.6% |
| Peru: 2000, 2013 | 87.6% | 89.2% |
| Bangladesh: 1999, 2013 | 78.8% | 81.6% |
|  | **Attendance at 4+ antenatal care visits** | |
| Ethiopia: 2000, 2014 | 9.7% | 32.1% |
| Kenya: 1998, 2014 | 60.4% | 57.6% |
| Malawi: 2000, 2014 | 55.4% | 44.7% |
| Niger: 1998, 2012 | 11.5% | 32.8% |
| Tanzania: 1999, 2010 | 68.8% | 42.8% |
| Peru: 2000, 2014 | 69.1% | 94.7% |
| Bangladesh: 1999, 2013 | 10.5% | 31.2% |
|  | **Skilled birth attendance** | |
| Ethiopia: 2000, 2014 | 5.6% | 15.5% |
| Kenya: 1998, 2014 | 44.4% | 61.8% |
| Malawi: 2000, 2014 | 54.4% | 87.4% |
| Niger: 1998, 2012 | 17.6% | 29.3% |
| Tanzania: 1999, 2010 | 42.9% | 48.9% |
| Afghanistan: 2003, 2011 | 14.3% | 38.6% |
| Peru: 2000, 2014 | 61.6% | 90.0% |
| Bangladesh: 1999, 2014 | 12.1% | 42.1% |
|  | **Exclusive breastfeeding of infants** | |
| Ethiopia: 2000, 2014 | 54.5% | 52.0% |
| Kenya: 1998, 2014 | 12.6% | 61.4% |
| Malawi: 2000, 2014 | 44.0% | 70.2% |
| Niger: 1998, 2012 | 0.8% | 23.3% |
| Tanzania: 1999, 2014 | 31.3% | 41.1% |
| Peru: 2000, 2014 | 66.9% | 68.4% |
| Bangladesh: 1999, 2014 | 46.4% | 55.3% |
|  | **DTP3 vaccination** | |
| Ethiopia: 2000, 2014 | 21.1% | 77.0% |
| Kenya: 1998, 2014 | 79.4% | 81.0% |
| Malawi: 2000, 2014 | 84.3% | 91.0% |
| Niger: 1998, 2014 | 25.1% | 68.0% |
| Tanzania: 1999, 2014 | 81.0% | 97.0% |
| Afghanistan: 2003, 2014 | 30.1% | 75.0% |
| Peru: 2000, 2010 | 84.8% | 88.0% |
| Bangladesh: 1999, 2014 | 72.1% | 95.0% |

**Table B.2-4: Levels in 1990 and 2013, and annual rates of change (ARC) over the full MDG era (1990-2013) as well as each decade within (1990-2000 and 2000-2013), for contextual variables, Countdown case study countries**

|  | China | Peru | Malawi | Bangladesh | Ethiopia | Niger | Tanzania | Kenya | Afghanistan | Pakistan |
| --- | --- | --- | --- | --- | --- | --- | --- | --- | --- | --- |
| **HDI** | | | | | | | | | | |
| 1990 level | 0.50 | 0.62 | 0.30 | 0.36 | 0.25 | 0.20 | 0.35 | 0.46 | 0.25 | 0.38 |
| 2013 level | 0.70 | 0.74 | 0.42 | 0.52 | 0.40 | 0.30 | 0.48 | 0.52 | 0.37 | 0.52 |
| *1990-2013 ARC* | *1.5%* | *0.8%* | *1.5%* | *1.6%* | *2.0%* | *1.9%* | *1.3%* | *0.5%* | *1.8%* | *1.3%* |
| *1990-2000 ARC* | *1.8%* | *0.9%* | *1.8%* | *1.8%* | *0.8%* | *1.7%* | *0.4%* | *-0.4%* | *-0.4%* | *0.9%* |
| *2000-2013 ARC* | *1.3%* | *0.7%* | *1.3%* | *1.3%* | *2.8%* | *2.0%* | *2.0%* | *1.2%* | *3.6%* | *1.6%* |
| **Access to improved sanitation** | | | | | | | | | | |
| 1990 level | 23.7% | 54.3% | 38.5% | 37.7% | 2.3% | 4.3% | 6.6% | 24.6% | 20.9% | 26.8% |
| 2013 level | 65.1% | 71.6% | 52.9% | 54.7% | 20.7% | 9.6% | 11.9% | 29.4% | 28.5% | 47.4% |
| *1990-2013 ARC* | *4.5%* | *1.2%* | *1.4%* | *1.6%* | *10.0%* | *3.6%* | *2.6%* | *0.8%* | *1.3%* | *2.5%* |
| *1990-2000 ARC* | *6.5%* | *1.5%* | *1.7%* | *1.9%* | *13.3%* | *5.1%* | *2.9%* | *0.9%* | *1.0%* | *3.4%* |
| *2000-2013 ARC* | *2.9%* | *1.0%* | *1.2%* | *1.5%* | *7.5%* | *2.4%* | *2.3%* | *0.7%* | *1.6%* | *1.8%* |
| **Access to safe water** | | | | | | | | | | |
| 1990 level | 66.7% | 74.8% | 41.6% | 76.2% | 13.6% | 34.7% | 55.0% | 42.7% | 4.8% | 85.3% |
| 2013 level | 91.7% | 85.3% | 83.7% | 83.2% | 49.0% | 50.3% | 53.3% | 60.9% | 60.6% | 91.4% |
| *1990-2013 ARC* | *1.4%* | *0.6%* | *3.1%* | *0.4%* | *5.7%* | *1.6%* | *-0.1%* | *1.6%* | *11.6%* | *0.3%* |
| *1990-2000 ARC* | *1.8%* | *0.7%* | *4.1%* | *0.4%* | *7.8%* | *2.0%* | *-0.1%* | *1.9%* | *16.4%* | *0.3%* |
| *2000-2013 ARC* | *1.0%* | *0.5%* | *2.3%* | *0.4%* | *4.1%* | *1.4%* | *-0.1%* | *1.3%* | *8.1%* | *0.3%* |
| **Gini** | | | | | | | | | | |
| 1990 level | 32.4 | 45.3 | 50.3 | 28.4 | 37.1 | 36.1 | 33.8 | 57.5 | 27.8 | 33.3 |
| 2013 level | 42.1 | 48.1 | 43.9 | 32.1 | 33.6 | 34.6 | 37.6 | 47.7 | 27.8 | 30.0 |
| *1990-2013 ARC* | *1.1%* | *0.3%* | *-0.6%* | *0.5%* | *-0.4%* | *-0.2%* | *0.5%* | *-0.8%* | *n/a* | *-0.4%* |
| *1990-2000 ARC* | *2.2%* | *1.1%* | *-0.8%* | *1.6%* | *-2.1%* | *1.7%* | *0.2%* | *-2.5%* | *n/a* | *-0.3%* |
| *2000-2013 ARC* | *0.3%* | *-0.4%* | *-0.4%* | *-0.3%* | *0.9%* | *-1.6%* | *0.6%* | *0.5%* | *n/a* | *-0.5%* |
| **Urbanisation** | | | | | | | | | | |
| 1990 level | 26.4% | 68.9% | 11.6% | 19.8% | 12.6% | 15.4% | 18.9% | 16.7% | 18.2% | 30.6% |
| 2013 level | 51.9% | 77.6% | 15.8% | 28.9% | 17.2% | 18.1% | 27.2% | 24.4% | 23.8% | 36.5% |
| *1990-2013 ARC* | *3.0%* | *0.5%* | *1.4%* | *1.6%* | *1.4%* | *0.7%* | *1.6%* | *1.6%* | *1.2%* | *0.8%* |
| *1990-2000 ARC* | *3.1%* | *0.6%* | *2.4%* | *1.8%* | *1.6%* | *0.5%* | *1.7%* | *1.7%* | *1.3%* | *0.8%* |
| *2000-2013 ARC* | *2.9%* | *0.5%* | *0.6%* | *1.6%* | *1.2%* | *0.9%* | *1.5%* | *1.6%* | *1.1%* | *0.7%* |
| **GNI** | | | | | | | | | | |
| 1990 level | 800 | 3120 | 400 | 540 | 390 | 500 | 590 | 980 | 499 | 1230 |
| 2013 level | 9210 | 10240 | 880 | 2070 | 1140 | 650 | 1590 | 1760 | 1627 | 3030 |
| *1990-2013 ARC* | *11.2%* | *5.3%* | *3.5%* | *6.0%* | *4.8%* | *1.1%* | *4.4%* | *2.6%* | *5.3%* | *4.0%* |
| *1990-2000 ARC* | *11.3%* | *4.3%* | *3.8%* | *4.9%* | *1.7%* | *0.4%* | *2.7%* | *1.4%* | *-1.4%* | *2.8%* |
| *2000-2013 ARC* | *11.1%* | *6.1%* | *3.3%* | *6.9%* | *7.2%* | *1.7%* | *5.7%* | *3.5%* | *10.7%* | *4.9%* |
| **Mean years of schooling** | | | | | | | | | | |
| 1990 level | 4.9 | 6.6 | 2.5 | 2.9 | 0.9 | 0.7 | 3.6 | 4.2 | 1.5 | 2.3 |
| 2013 level | 7.5 | 8.7 | 4.2 | 4.8 | 2.2 | 1.4 | 5.1 | 7.0 | 3.1 | 4.9 |
| *1990-2013 ARC* | *1.9%* | *1.2%* | *2.3%* | *2.2%* | *3.9%* | *3.1%* | *1.5%* | *2.2%* | *3.2%* | *3.3%* |
| *1990-2000 ARC* | *3.0%* | *1.6%* | *1.8%* | *2.5%* | *5.1%* | *4.6%* | *2.5%* | *3.5%* | *3.4%* | *3.7%* |
| *2000-2013 ARC* | *1.0%* | *0.9%* | *2.6%* | *2.0%* | *3.0%* | *1.9%* | *0.8%* | *1.3%* | *3.0%* | *3.1%* |
| **Female literacy** | | | | | | | | | | |
| 1990 level | 68.1% | 79.7% | 39.1% | 25.1% | 18.5% | 9.4% | 50.1% | 77.9% | 47.0% | 22.3% |
| 2013 level | 91.3% | 84.6% | 68.5% | 52.2% | 28.9% | 15.1% | 67.5% | 84.2% | 66.6% | 40.3% |
| *1990-2013 ARC* | *1.3%* | *0.3%* | *2.5%* | *3.2%* | *2.0%* | *2.1%* | *1.3%* | *0.3%* | *1.5%* | *2.6%* |
| *1990-2000 ARC* | *2.4%* | *0.3%* | *3.7%* | *4.6%* | *1.3%* | *0.0%* | *1.8%* | *0.0%* | *1.7%* | *3.3%* |
| *2000-2013 ARC* | *0.4%* | *0.3%* | *1.5%* | *2.2%* | *2.5%* | *3.7%* | *0.9%* | *0.6%* | *1.4%* | *2.1%* |

*n/a indicates that annual change was not computed because there were not 2 values available over this time period*

# C. References

1. Barros AJ, Ronsmans C, Axelson H, Loaiza E, Bertoldi AD, França GV, Bryce J, Boerma JT, Victora CG: **Equity in maternal, newborn, and child health interventions in Countdown to 2015: a retrospective review of survey data from 54 countries**. *The Lancet* 2012, **379**(9822):1225-1233.

2. Victora CG, Wagstaff A, Schellenberg JA, Gwatkin D, Claeson M, Habicht J-P: **Applying an equity lens to child health and mortality: more of the same is not enough**. *The Lancet* 2003, **362**(9379):233-241.

3. Singh NS, Huicho L, Afnan-Holmes H, John T, Moran AC, Colbourn T, Grundy C, Matthews Z, Maliqi B, Matthews M *et al*: **Countdown to 2015 country case studies: Systematic tools to address the “black box” of health systems and policy assessment**. *BMC Public Health* 2016, **(in press)**.

4. Mann C, Ng C, Akseer N, Bhutta ZA, Borghi J, Colbourn T, Hernandez-Pena P, Malik MA, Martinez-Alvarez M, Ahmad SS *et al*: **Countdown to 2015 country case studies: What can analysis of national health financing contribute to understanding MDG 4 and 5 progress?** *BMC Public Health* 2016 (**in Press**).

5. Walker N, Tam Y, Friberg I: **Overview of the Lives Saved Tool (LiST)**. *BMC Public Health* 2013, **13**(Suppl 3):S1.

6. Winfrey W, McKinnon R, Stover J: **Methods used in the lives saved tool (LiST)**. *BMC Public Health* 2011, **11**(Suppl 3):S32.

7. Rudan I, Gibson JL, Ameratunga S, El Arifeen S, Bhutta ZA, Black M, Black RE, Brown KH, Campbell H, Carneiro I *et al*: **Setting priorities in global child health research investments: guidelines for implementation of CHNRI method**. *Croat Med J* 2008, **49**(6):720-733.

8. Barros AJD, Victora CG: **Measuring Coverage in MNCH: Determining and Interpreting Inequalities in Coverage of Maternal, Newborn, and Child Health Interventions**. *PLoS Med* 2013, **10**(5):e1001390.

9. Kerber KJ, de Graft-Johnson JE, Bhutta ZA, Okong P, Starrs A, Lawn JE: **Continuum of care for maternal, newborn, and child health: from slogan to service delivery**. *The Lancet* 2007, **370**(9595):1358-1369.

10. UNICEF, WHO, World Bank, Nations U: **Levels & Trends in Child Mortality**. In*.*; 2015.

11. United Nations Department of Economic and Social Affairs - Population Division: **World Population Prospects: The 2015 Revision**. In*.*; 2015.

12. United Nations Department of Economic and Social Affairs - Population Division: **World Population Prospects: The 2010 Revision**. In*.*; 2011.

13. World Health Organization, UNICEF: **Accountability for Maternal, Newborn & Child Survival: The 2013 Update** In*.*; 2013.

14. Huicho L, Segur E, Huayanay C, Niño de Guzman J, Restrepo-Méndez MC, Tam Y, Barros AJD, Victora CG, Group tPCCCSW: **Placing child health and nutrition at the centre of the antipoverty political agenda: the Peruvian experience, 2000-2013**. In*.*; 2015.

15. Kanyuka M, Ndawala J, Mleme T, Chisesa L, Makwemba M, Amouzou A, Borghi J, Daire J, Ferabee R, Hazel E *et al*: **Malawi and Millennium Development Goal 4: Early Adopter; Early Achiever**. In*.*; 2015.

16. Afnan-Holmes H, Magoma M, John T, Levira F, Msemo G, Armstrong CE, Martinez-Alvarez M, Kerber K, Kihinga C, Makuwani A *et al*: **Tanzania's Countdown to 2015: an analysis of two decades of progress and gaps for reproductive, maternal, newborn, and child health, to inform priorities for post-2015**. *The Lancet Global health* 2015, **3**(7):e396-409.

17. Ethiopian Public Health Institute (EPHI): **Countdown to 2015: Ethiopia's Progress Towards Reduction in Under-Five Child Mortality**. In*.*; 2015.

18. Bhatti Z, Hafiz A, Sattar Z, Hazir T, Rizvi A, Malik A, Bhutta Z: **Countdown to 2015 In-Depth Case Study: Pakistan**. In: *Case Study Workshop.* London UK; 2015.

19. **Health statistics and information systems**

20. World Health Organization, UNICEF, UNFPA, The World Bank, United Nations: **Trends in maternal mortality: 1990 to 2015**; 2015.
